# New insights on *Pseudoalteromonas haloplanktis* TAC125 genome organization and benchmarks of genome assembly applications using next and third generation sequencing technologies

**DOI:** 10.1038/s41598-019-52832-z

**Published:** 2019-11-11

**Authors:** Weihong Qi, Andrea Colarusso, Miriam Olombrada, Ermenegilda Parrilli, Andrea Patrignani, Maria Luisa Tutino, Macarena Toll-Riera

**Affiliations:** 1 Functional Genomics Center Zurich, ETH Zürich/University of Zurich, Winterthurerstrasse 190, 8057 Zurich, Switzerland; 2 Department of Chemical Sciences, Federico II University of Naples, Complesso Universitario Monte Sant’Angelo, via Cintia, I-80125 Naples, Italy; 3 Department of Evolutionary Biology and Environmental Studies, University of Zurich, Winterthurerstrasse 190, 8057 Zurich, Switzerland; 4 Swiss Institute of Bioinformatics, Quartier Sorge-Bâtiment Génopode, Lausanne, 1015 Switzerland

**Keywords:** High-throughput screening, Sequence annotation, Bacterial genomics, Marine microbiology, Bacterial genes

## Abstract

*Pseudoalteromonas haloplanktis* TAC125 is among the most commonly studied bacteria adapted to cold environments. Aside from its ecological relevance, *P. haloplanktis* has a potential use for biotechnological applications. Due to its importance, we decided to take advantage of next generation sequencing (Illumina) and third generation sequencing (PacBio and Oxford Nanopore) technologies to resequence its genome. The availability of a reference genome, obtained using whole genome shotgun sequencing, allowed us to study and compare the results obtained by the different technologies and draw useful conclusions for future *de novo* genome assembly projects. We found that assembly polishing using Illumina reads is needed to achieve a consensus accuracy over 99.9% when using Oxford Nanopore sequencing, but not in PacBio sequencing. However, the dependency of consensus accuracy on coverage is lower in Oxford Nanopore than in PacBio, suggesting that a cost-effective solution might be the use of low coverage Oxford Nanopore sequencing together with Illumina reads. Despite the differences in consensus accuracy, all sequencing technologies revealed the presence of a large plasmid, pMEGA, which was undiscovered until now. Among the most interesting features of pMEGA is the presence of a putative error-prone polymerase regulated through the SOS response. Aside from the characterization of the newly discovered plasmid, we confirmed the sequence of the small plasmid pMtBL and uncovered the presence of a potential partitioning system. Crucially, this study shows that the combination of next and third generation sequencing technologies give us an unprecedented opportunity to characterize our bacterial model organisms at a very detailed level.

## Introduction

Cold environments covering most of the Earth, harbour a vast diversity of cold-adapted organisms^1^. *Pseudoalteromonas haloplanktis* TAC125 is among the most well studied. *P. haloplanktis* TAC125 is a fast growing gamma-proteobacterium isolated from Antarctic coastal seawater^2^ that can survive in temperatures ranging from −2,5 °C to 29 °C^3,4^. Its genome has been fully sequenced using whole genome shotgun methodology, identifying two chromosomes. Interestingly, a significant fraction of chromosome II, the smallest chromosome, shows similarity to genes typically encoded in plasmids, suggesting that chromosome II has its origin in a plasmid^2^. Additionally, *P. haloplanktis* TAC125 harbours a small cryptic plasmid, pMtBL^5^. Besides the characterization of *P. haloplanktis*’ genome, other aspects have been studied in detail, such as its growth in different media^4,6–8^, biofilm formation^9^ and proteome expression at different temperatures^3,10,11^. Moreover, genetic tools to manipulate *P. haloplanktis’* genome^12–15^ and a metabolic model have been previously described^7,16^. Aside from being a relatively well-studied and characterized cold-adapted bacterium, it has significance for biotechnological applications; it has been used for the production of recombinant proteins that are difficult to produce in commonly used expression hosts^4,17–23^ and its potential use for bioremediation has been suggested^24^.

The method applied to generate the *P. haloplanktis* TAC125 reference genome, whole genome shotgun sequencing using Sanger sequencing technology, has enabled the sequencing of many genomes, including those of human and mouse. However it is expensive, labour-intensive and time-consuming^25^. Conversely, Next Generation Sequencing (NGS) technologies, using massively parallel processing, brought the cost down significantly and dramatically reduced the sequencing time. But NGS reads are shorter, thus tend to yield more fragmented genome assemblies^26^. Third generation sequencing technologies, such as Oxford Nanopore Technologies (ONT) real-time direct DNA/RNA sequencing and Pacific Biosciences (PacBio) Single Molecule, Real-Time (SMRT) Sequencing, can produce extremely long reads (20 kb and even longer) and therefore are more suitable for generating highly continuous genomes^27,28^. These technologies open new doors in microbial genomics and enable a broad range of microbial studies^29^. Due to the importance of *P. haloplanktis* TAC125 as a model organism for cold-adaptation and its importance for biotechnological applications, it is key to reanalyse its genomic asset using these newer sequencing technologies.

In this study we resequenced *P. haloplanktis* TAC125 genome using NGS (Illumina) and the two third generation sequencing methods (ONT and PacBio). The resequencing efforts not only identified one misassembled tandem repeat of 1.2 kb in the reference chromosome NC_007481.1, but also revealed the presence of a large plasmid that was unnoticed in the first genome sequence^2^. Besides the annotation and analysis of the newly identified plasmid (pMEGA), we further characterized the already described plasmid (pMtBL) identifying a putative plasmid segregation system. The available reference genome sequences thereby and high coverage long read data also allowed us to generate accurate and realistic measures of advantages and limitations of these newer sequencing technologies for *de novo* genome sequencing applications, and how they were affected by sequencing depth. The findings can be instrumental for all researchers who are planning *de novo* genome sequencing projects using these newer technologies.

## Results

### Sequencing and assembly of the *P. haloplanktis* TAC125 genome

We resequenced *P. haloplanktis* TAC125 genome using Illumina, ONT and PacBio technologies with high coverage (195X–573X) (Table 1). The ONT sequencing library was prepared without size selection and produced much longer reads than the size selected PacBio library. The N50 value of ONT reads reached 24 kb and the longest ONT read arrived at 183 kb. The N50 value of PacBio reads was about 12 kb, consistent with the size selected during the sequencing library preparation. Compared to the PacBio reads, ONT reads had slightly higher average base qualities, but the reported quality scores were also more variable (Supplementary Fig. S1). Since the base quality scores are specific to sequencing vendors and their chemistry, the values cannot be compared directly across the technologies^30^. We thus aligned the reads to the reference genome (NC_007481.1, NC_007482.1) and evaluated the read quality based on sequence alignments. Within the aligned regions, ONT reads did show lower alignment error rate, but they also had lower mapping rate with higher fraction of reads containing unaligned regions that were clipped away, which might be due to the high variability of base qualities along each read and within the full dataset. For both ONT and PacBio, the reported average base quality score was more or less consistent with the Phred score calculated from the alignment error rate (−10*log(alignment_error_rate,10)). For Illumina, the reported average quality score was 39, representing a 0.1% error rate. The alignment error rate was around 0.2%, equivalent to a quality score of 27. Alignment error rate was much higher for ONT and PacBIO, 11.19% and 17.72% respectively. Read alignment also revealed that over 90% of both PacBio and ONT reads harboured at least one insertion and/or deletion, while only 0.03%-0.06% of the Illumina reads had InDel errors. PacBio reads contained more insertion than deletion errors, while ONT reads showed the opposite trend.

**Table 1:** Sequencing output metrics.

	ONT	PacBio	Illumina
Input DNA	HMW DNA, without shearing	HMW DNA, sheared and size selected for fragments longer than 10 kb	DNA was isolated using the DNeasy Blood & Tissue kit (QIAGEN)
Library preparation kit	ONT 1D ligation sequencing kit	PacBio P6 DNA/Polymerase binding kit 2.0	Illumina TruSeq
Sequencer	GridIon X5	PacBio RSII	HiSeq. 4000
Run time	24 hours	6 hours	3.5 days
Num. reads	194,538	92,873	3,452,040
Num. bases (bp)	2,293,338,560	779,603,216	1,035,612,000
Read N50 (bp)	23,927	12,153	2 × 150
Longest read (bp)	183,036	69,046	2 × 150
Mean read length (bp)	11,789	8,394	2 × 150
Estimated coverage^a^	573 X	195 X	259 X
Average Phred quality	9.9	8.2	39
General alignment error rate^b^	11.19%	17.72%	0.2%
Insertions	40,665,761	46,593,977	2,014
Mapped reads with at least one insertion	97.89%	93.35%	0.03%
Deletions	51,868,727	19,348,548	3,757
Mapped reads with at least one deletion	97.95%	93.01%	0.06%
Mapped reads	88.65%	95.17%	97.76
Clipped mapped reads	86.86%	83.36%	0.39%

^a^Assuming a genome size of 4 Mb.

^b^Computed as a ratio of total collected edit distance to the number of mapped bases.

With both PacBio and ONT data, chromosome level assemblies were achieved and the two reference chromosomes were 100% covered by the assembled contigs. Illumina data yielded less continuous and complete genome drafts (Table 2). Although both the ONT and PacBio assemblies were highly continuous and complete, they differed at consensus accuracy. After sequencer-specific error correction (see Materials and Methods), there were still a few thousand InDel and base substitution errors remaining in the ONT assembly, which were further removed with Illumina reads. In the PacBio assembly, Illumina reads only removed a few hundred InDels. Among the InDels and substitutions remaining in the final genome drafts, 12 SNPs and 5 InDels were common in the genome drafts from all three technologies (Supplementary Fig. S2, Supplementary Table S1). If assuming the reference sequences were 100% accurate, the final consensus accuracy of PacBio contigs reached 99.99%, which is comparable to the accuracy of Illumina contigs. The final consensus accuracy of the ONT contigs was still slightly lower (99.98%), with over 100 InDel and substitution errors remaining.

**Table 2:** Assembly statistics of circularized, trimmed and polished genome drafts.

	ONT	PacBio		Illumina
Assembler	Canu	HGAP3	Canu	SPAdes
Circularizing and trimming	amos, minimus2	amos, minimus2	amos, minimus2	NA
Aligner	bwa	blasr	blasr	NA
Sequencer-specific consensus polishing	Nanopolish	Quiver	Quiver	NA
Polishing using Illumina reads	Pilon	Pilon	Pilon	NA
Num. Contigs	3	3	3	109
Total Length (bp)	3,996,798	3,940,687	3,913,837	3,883,161
N50 (bp)	3,295,052	3,240,603	3,213,753	414,366
GC%	40.07	40.07	40.07	39.97
Num. substitution errors corrected using Illumina reads	2,069	0	0	NA
Num. InDel errors corrected using Illumina reads	3,253	376	386	NA
Reference genome coverage (%)	100	100	100	98.89
Average identity to the reference genome (%)	99.98	99.99	99.99	99.99
Num. residual SNPs	53	40	40	34
Num. residual InDels	87	24	24	28
Miss assemblies*	2	1	1	0
Num. Ns	82	0	0	0

^*^One reported miss assembly is actually due to an assembly error in the reference genome (Supplementary Fig. S3).

Resequencing of the genome using newer technologies identified one miss assembly event in the reference genome, where the sequence between 2,064,625 and 2,065,827 was wrongly assembled twice and formed a tandem repeat (Supplementary Fig. S3).

When comparing against the reference genome, it was also found that the 1,644 bp sequence between 560,857 and 562,502 on chromosome NC_007482.1 was assembled tandemly in the ONT contig (tig00000003:277,635-280,934), with 10 ambiguous bases (NNNNNNNNNN) inserted in between (tig00000003:279,279-279,290; Supplementary Fig. S4). This tandem repeat was not observed with either the PacBio or the Illumina contig (not shown) and there were no PacBio long reads aligned across the 10 bp ambiguous sequence (Supplementary Fig. S4). The Ns were introduced during circularization by minimus2 due to sequence variation at those sites within overlapped contig ends. When PacBio data was assembled using the same assembler, Canu, the error was not reproduced (Table 2), thus it was likely due to errors carried from ONT reads that hindered the contig circularization process.

Genome resequencing using the newer technologies also identified a novel plasmid, pMEGA. All three genome drafts harboured a novel contig over 63 kb long (Supplementary Table S2). The corresponding contig in the ONT genome draft was one base shorter and 99.96% identical to the PacBio pMEGA contig. The minor sequence differences between the PacBio and ONT pMEGA sequences were mainly due to uncalled bases remaining in the ONT contig and three single base InDels. The Illumina pMEGA contig was 1.2 kb shorter (Supplementary Fig. S5a) but the rest of the sequences were identical to the PacBio contig sequences. A search among all Illumina contigs using PacBio pMEGA sequence showed that this region (pMEGA:8149-9494) was actually tiled by five short Illumina contigs that ranged from 200 to 477 bp with 100% sequence similarity. Alignment of PacBio reads against the PacBio pMEGA contig revealed the presence of long reads aligning across the 1.2 kb region (Supplementary Fig. S5b) This region shared 97% sequence similarity with chromosome NC_007482.1. Apparently the repetitiveness was only resolvable with the help of long reads. Since the PacBio contig was the most complete and continuous, with accuracy identical to Illumina assembled sequences, it was selected as the pMEGA sequence reported in this study.

### Influence of long read sequencing depth on assembly outcomes

Compared to Illumina sequencing, ONT and PacBio sequencing are still more expensive in terms of cost per base. Most genome drafts based on long reads have coverage lower than 100X, and residual errors in the genome drafts could hinder accurate functional annotation^31^. In our study we observed that although our ONT reads had the highest sequencing depth, they still yielded less accurate consensus sequences after assembly and polishing using both ONT and Illumina data. To further understand this observation we decided to study how the change of sequencing depth influenced the long read assembly outcomes. We sub-sampled the ONT and PacBio reads to 25X, 50X, 100X and 200X, respectively. The sub-sampled reads were assembled using Canu, corrected with Illumina data, and compared against the reference genome for the measurement of the final consensus accuracy.

**Figure 1:**
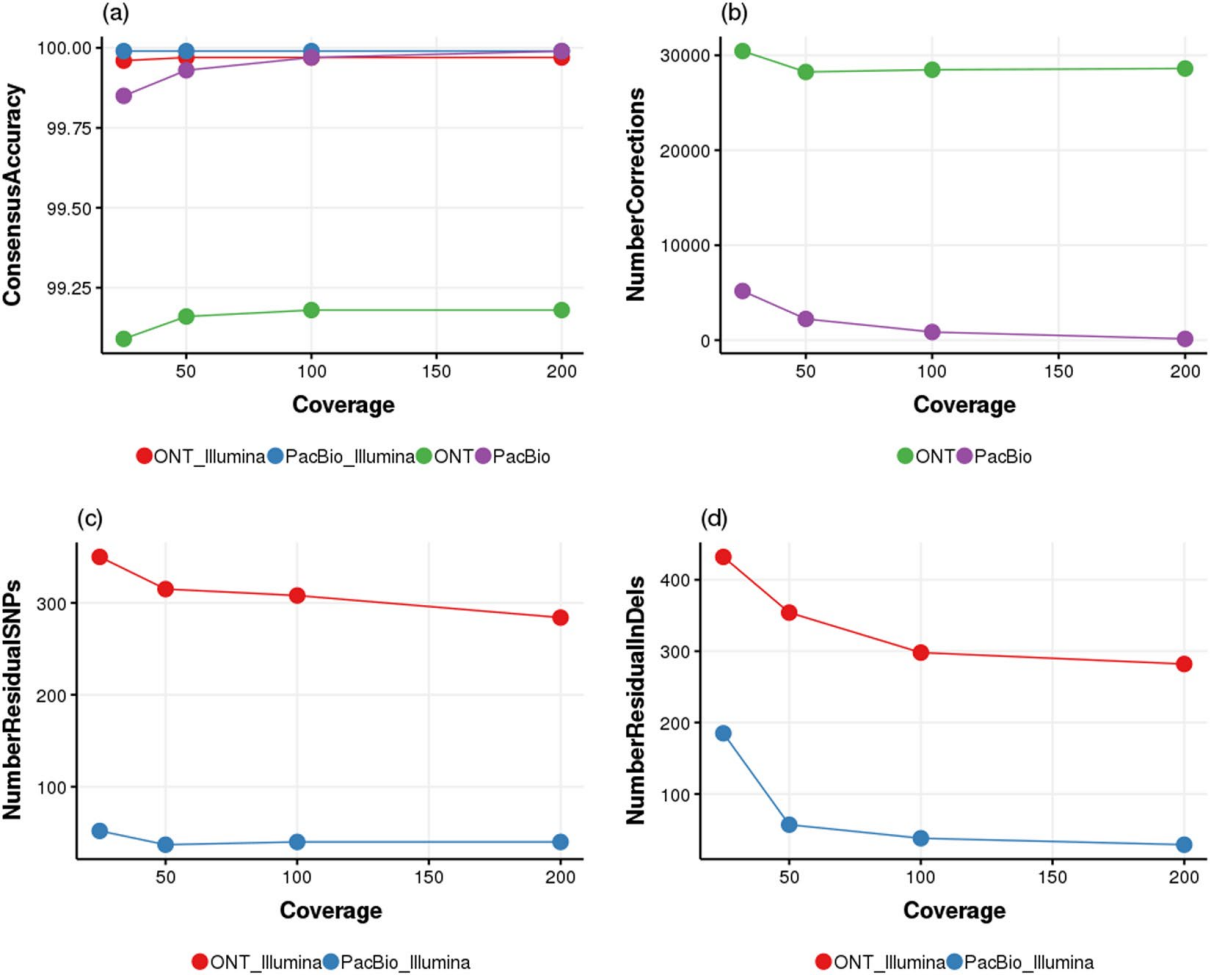
Effects of sequencing coverage on the consensus accuracy of Canu assemblies of ONT and PacBio reads.

For the PacBio dataset, when the sequence depth was only 25X, a more fragmented assembly was produced (Supplementary Table S3). But with a sequencing depth of 50X and above, chromosome level assembly (Fig. 1b) was consistently achieved. For the ONT dataset, even with 25X sequencing depth, the two reference chromosomes were assembled completely into two contigs, although pMEGA was only partially (90%) reconstructed into two individual contigs. It suggested that the longer ONT read length (Table 1) helped to improve the assembly continuity, even at low sequencing depth. With increasing sequencing depth, chromosome level assemblies of the reference chromosomes and pMEGA were consistently achieved with ONT reads as well, but the number of contigs varied from 3 to 6, not correlating with sequencing depth (Supplementary Table S3). The extra contigs were found out to be shorter products that partially covered either the reference chromosomes or the pMEGA sequence. We hypothesize that these assembly artefacts could be caused by the higher quality variation observed among ONT reads. To confirm this we filtered out ONT reads with mean quality scores lower than 7 and ran the simulation again. With filtered ONT reads, chromosome level assemblies were achieved consistently with coverage 25X and above (Supplementary Table S3). This observation suggests that filtering out low quality ONT reads was important for removing assembly artefacts and increasing assembly continuity at low coverage, although filtering of ONT reads by mean quality score was shown to be less crucial on read alignment rate^32^.

At all simulated sequencing depth, the PacBio contigs were more accurate than the ONT contigs (Fig. 1a). Before polishing using Illumina data, with 50X, 100X and 200X PacBio read coverage, the consensus accuracy increased steadily, and reached 99.93%, 99.97% and 99.99%, respectively. Higher PacBio read coverage was mainly helpful in removing InDels (Fig. 1d). For the ONT dataset the sequencing depth had less effect on the final consensus accuracy, especially when the coverage was higher than 100X. The corresponding accuracy for ONT contigs at 50X, 100X and 200X coverage was only 99.16%, 99.18% and 99.18%, respectively. Higher ONT read coverage was helpful for decreasing both InDel and substitution errors (Fig. 1c,d). Due to the big difference on the initial consensus accuracy, Illumina data were able to correct much more errors (Fig. 1b) in ONT contigs than in PacBio contigs. The polished contigs reached the same consensus accuracy regardless of the initial long read coverage, 99.99% for PacBio contigs and 99.97% for ONT contigs.

The effect of ONT read quality on the consensus accuracy was coverage dependent. When the coverage was higher than 100X low quality reads did not affect the consensus accuracy. With coverage lower than 100X filtering out the low quality reads helped to slightly increase the initial consensus accuracy (Supplementary Fig. S6), but such pre-processing did not increase the final consensus accuracy (99.97%) after polishing with Illumina data.

For a fair comparison, we removed the sequencer specific long read polishing step in this simulation study. When taking this step into account, the final consensus accuracy of ONT contigs was further improved. In comparison to polishing using only Illumina reads, polishing using both Illumina and ONT reads helped to remove 224 more SNPs and 150 more InDels (Supplementary Table S4). For PacBio contigs, polishing using both Illumina and PacBio reads helped to remove three more InDels in the final consensus, which Illumina data alone did not manage to correct.

### Analysis of pMEGA

**Figure 2:**
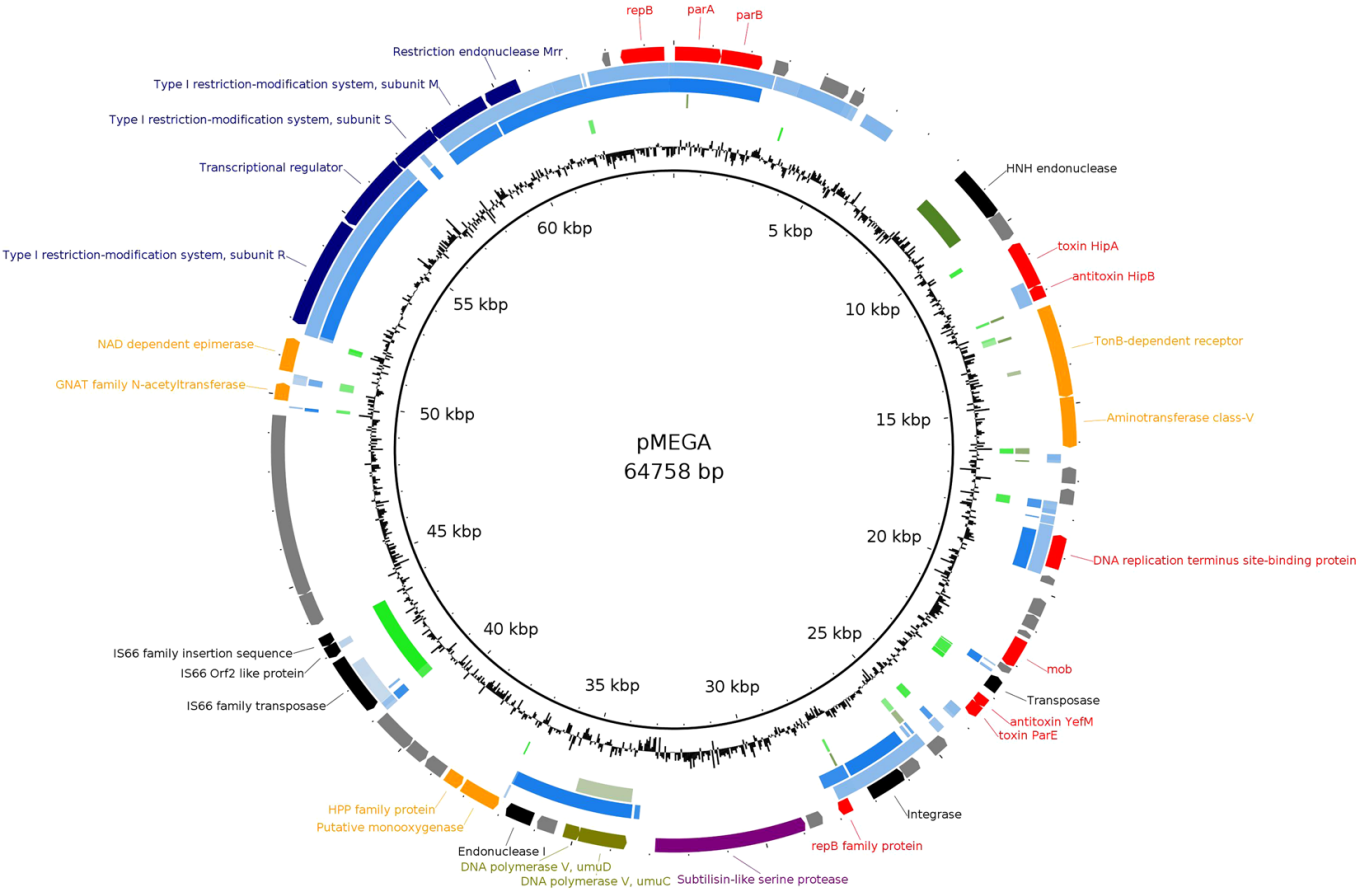
Schematic representation of pMEGA. Genes are depicted as arrows in the outermost circle; the arrowhead indicates the direction of transcription. Arrows coloured in red are involved in plasmid housekeeping functions (replication, partition, stability). Black arrows indicate genes involved in DNA rearrangements, orange arrows genes involved in metabolic functions, in navy blue defence genes, in green genes involved in mutagenesis, in purple in proteolysis and grey indicates genes with unknown function. The second outer circle depicts homology to *Pseudoalteromonas arctica* plasmid (>50% nucleotide identity); the third circle indicates homology to *Pseudoalteromonas nigrifaciens* plasmid (>50% nucleotide identity). The fourth and the fifth circle indicate homology to *P. haloplanktis* TAC125 chromosome I and II, respectively (>50% nucleotide identity). The intensity of the colour indicates the % of nucleotide identity, the more intense the colour is, the higher the % of identity is. The innermost circle represents the GC content.

As mentioned above, the resequencing of *P. haloplanktis* TAC125 using third generation sequencing technologies revealed the presence of a novel plasmid, pMEGA. pMEGA has 64,758 bp, contains 52 open reading frames (ORFs) and has a GC content of 38.61% (Fig. 2). The GC content is marginally lower than the GC content in *P. haloplanktis* TAC125 chromosome I and II (41% and 39.3%, respectively^2^). It is found to be a low-copy number plasmid; the plasmid copy number (PCN) estimated by qPCR is 0.86 ± 0.18 and 0.97 ± 0.20 in mid and late-exponential phases, respectively. It is a non-conjugative plasmid, as no conjugation genes could be identified. However, two lines of evidence suggest that this is a putatively mobilizable plasmid. First, PSHA_p00019 shows homology to the Pfam protein family plasmid recombination enzyme (PF01076). Second, OriTfinder^33^ identifies PSHA_p00019 as being a relaxase. Relaxases are enzymes that nick at the origin of transfer (*oriT*) and prepare the plasmid for its mobilization by conjugation^34^. Bacterial mobilization systems have been classified in 6 different types, and the putative relaxase found in pMEGA has homology to MOB_V_ family^34^. However, neither OriTfinder^33^, PlasmidFinder^35^ nor manual searches could identify the *oriT* required for mobilization. The OriTfinder and PlasmidFinder databases do not contain *oriT* from marine bacteria, which limits the power to identify an *oriT* in pMEGA.

pMEGA has a RepB family replication protein and a type Ia partitioning system composed of ParA and ParB proteins. Additionally, pMEGA maintenance and stability is mediated by two type II toxin-antitoxin systems, the HipBA system and the hybrid yefM-ParE system^36^. Proteins encoded in pMEGA can be classified into 6 functional categories (Fig. 2). The two most abundant functional categories correspond to proteins involved in plasmid housekeeping functions (replication, partition and stability) and mobilization of transposable elements (integrases, transposases and endonucleases), with 10 and 7 proteins, respectively. We also found several proteins with a role in metabolism, such as TonB-dependent receptor, an aminotransferase, a nitronate monooxygenase, an epimerase and an acetyltransferase. Indeed, amino acid metabolism has been suggested to be beneficial in the nutrient-limited cold-environments because amino acids can be used both as carbon and nitrogen sources^37^. Moreover, pMEGA hosts a subtilisin-like serine protease and also codes for two defence mechanisms against bacteriophages. It has a type I restriction-modification system, the most complex type of restriction-modification systems, which is composed of a restriction subunit, a specificity subunit and a modification subunit^38^. pMEGA also contains the simplest restriction-modification system, type IV, which only encodes a restriction endonuclease (Mrr in this case) that recognizes and cuts modified foreign DNA^39^. Remarkably, pMEGA also harbours an *umuDC* DNA repair operon, which codes for DNA polymerase V (DNA PolV). DNA PolV is a translesion synthesis polymerase that bypasses DNA damage facilitating replication, but because it is an error-prone polymerase, it is highly mutagenic^40^. We identified a LexA binding site (CACTGTATATATAAACAGTA) in the promoter region of DNA PolV suggesting that the repressor LexA, which represses SOS response genes, regulates its expression. Indeed, it is well known that DNA PolV is induced by the SOS response in the presence of DNA damage. No other LexA binding sites were found in pMEGA, but we identified 34 LexA binding sites in chromosome I and 5 in chromosome II.

pMEGA nucleotide similarity to *P. haloplanktis* TAC125 chromosomes is scarce, only 5% of pMEGA sequence has similarity to chromosome I and 2% has similarity to chromosome II (% of identity >85%) (Supplementary Table S5). Most of the similarities are in intergenic regions (some are annotated as pseudogenes in *P. haloplanktis* chromosomes) with the exception of two regions. The first region shows 97.6% identity to a IS679 insertion sequence found in chromosome I (PSHA_RS02020-PSHA_RS02030 genes) and the second region displays 96.7% identity to an HNH endonuclease encoded in chromosome II (PSHA_RS16255) (further details in Supplementary Text).

Nucleotide similarity searches against the NCBI nucleotide collection database (nr/nt) revealed the uniqueness of pMEGA (Fig. 2, Supplementary Tables S5 and S6). Only 2 hits covered more than one third of the pMEGA sequence, 36% being the maximum coverage. These two hits correspond to two plasmids harboured in two marine bacteria, *Pseudoalteromonas arctica* (strain A 37-1-2, CP011027.1) and *Pseudoalteromonas nigrifaciens* (strain KMM661, CP011038.1) isolated from the Arctic (Spitzbergen, Norway)^41^ and Japan, respectively. Out of the 52 ORFs identified in pMEGA, 20 are found in *P. arctica* and *P. nigrifaciens* with a % of identity of at least 90% and 8 additional ORFs share homology with *P. nigrifaciens* or *P. arctica* (Supplementary Text).

### Reannotation of pMtBL

pMtBL is a *P. haloplanktis* TAC125 small endogenous plasmid which was isolated in 2001^5^. In this study its sequence was confirmed with Illumina data, but it was missed in PacBio and ONT genome drafts. The DNA isolation protocol used to prepare the high molecular weight input DNA for PacBio and ONT sequencing might have filtered out the DNA of this small plasmid.

**Figure 3:**
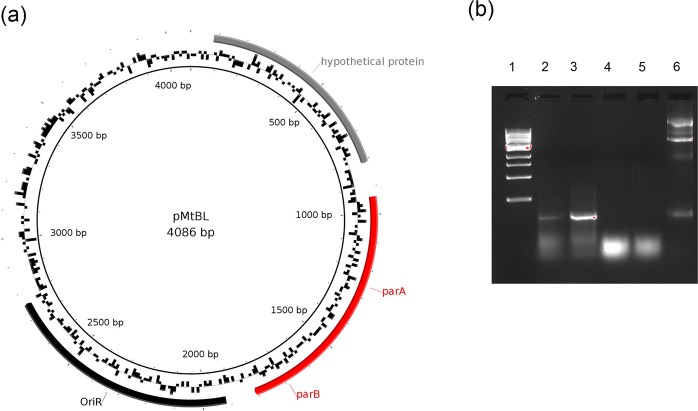
ORFs analysis of pMtBL. (**a**) pMtBL map. The *OriR* is highlighted in black. Manually analysed putative ORFs are represented as thick arrows. (**b**) *orf2* expression analysis using end-point RT-PCR. After total RNA extraction a cDNA was synthetized using the primer pMtBL_*B7*_rv specific for *orf2*. Then PCR reactions with primers pMtBL_*A4*_fw and pMtBL_*B7*_rv were performed on the cDNA obtained from total *P. haloplanktis* TAC125 RNA after growth in GG (lane 2) and TYP media (lane 3). The PCR reaction was also carried out directly on RNA extracted after growths in GG (lane 4) and TYP (lane 5) and on total *P. haloplanktis* TAC125 DNA (lane 6). The expected amplicon of <100 bp was obtained only in the reactions where either the cDNA (lanes 2 and 3) or the total bacterial DNA (lanes 6) were used as templates. Total RNA templates did not lead to any amplification demonstrating the absence of DNA cross-contamination (lanes 4 and 5). Lane 1, 1 kb NEB marker. Full-length gel is presented in Supplementary Fig. S7.

When pMtBL was discovered, a restriction analysis led to the isolation of its minimal replication origin (OriR), which was then used for the development of a series of shuttle vectors^4,5,13,19^. However, our qPCR analysis reveals that pMtBL exists as a single copy in *P. haloplanktis* TAC125, as indicated by the measured PCN values of 1.1 ± 0.09 in mid exponential phase and 1.34 ± 0.06 in late exponential phase. The stable inheritance of this low-copy number DNA molecule after cell division is probably assured by the existence of a plasmid segregation system, as suggested by a re-analysis of pMtBL sequence. A manually refined prediction of pMtBL ORFs suggests the presence of 3 putative encoding sequences (Fig. 3a). All three ORFs have homologues in other bacteria according to BLASTP searches against the non-redundant protein database from NCBI, although in all the cases they are predicted as hypothetical CDSs. Furthermore, both orf1 and orf3 are characterized by the presence of Shine Dalgarno (SD) consensus sequences upstream of the starting codon. Although orf2 does not possess a canonical SD, its expression was verified via RT-PCR with primers specific for the second half of the putative CDS (Fig. 3b).

A further *in silico* analysis indicated that orf2 probably encodes a Walker-type NTPase as the translated sequence harbours a P-loop motif (KGGXXK[TS]) at the N-terminal extremity^42^. Furthermore, the first 100 amino acids of ORF2 constitute a domain belonging to the cd02042 superfamily, whose main representative is *Caulobacter crescentus* ParA protein, according to the NCBI prediction tool. Considering this information and the close proximity of orf2 to the replication origin^43^, we can affirm that this gene is likely to encode a protein involved in plasmid segregation processes. If pMtBL orf2 was actually a *par*A gene, its genetic partners should probably be very close. Therefore, orf3 might be the *par*B gene, but the shortage of close homologues in the data banks makes it more difficult to label this putative gene.

**Figure 4:**
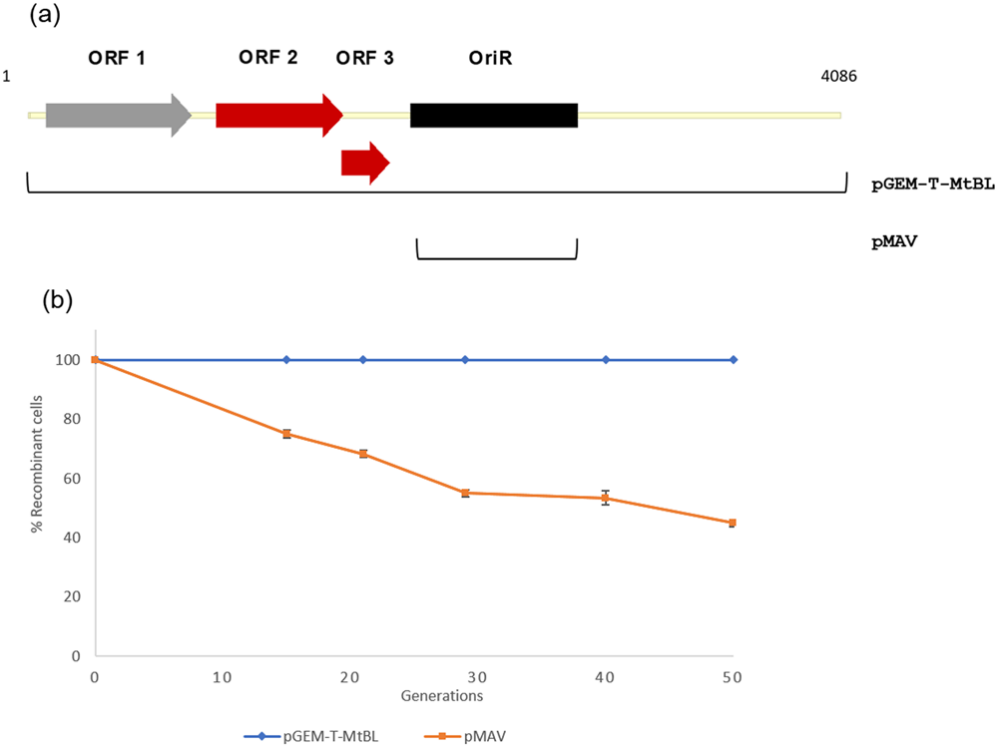
Schematic diagram of pMtBL derivative shuttle vectors and their segregational stability. (**a**) Overview of the extent of pMtBL regions included in each vector series. pGEM-T-MtBL encompasses the entire pMtBL plasmid; MAV was developed only introducing the psychrophilic OriR^4^. (**b**) Retention of plasmids representative of each family derived from pMtBL without antibiotic selection. Each experiment was carried out as biological duplicates.

To define if pMtBL regions other than its minimal replication origin could affect its segregational stability, we compared the loss rate of two pMtBL derived shuttle vectors. The first is pGEM-T-MtBL, which includes the whole sequence of pMtBL linearized with XbaI restriction site and fused with pJB3 OriT and pGEM4Z backbone. The second is pMAV and is a representative of CloneQ series^5^, which only possesses pMtBL OriR^4^. pMtBL regions included in each construct are schematized in Fig. 4a and cloning details are described in the Materials and Methods section. The segregational stability of the vectors was evaluated in absence of selective pressure in a *P. haloplanktis* TAC125 cured strain named KrPl devoid of the endogenous pMtBL plasmid (personal communication). This analysis revealed that the single presence of the minimum psychrophilic OriR does not guarantee the complete preservation of the plasmid in Krpl. While pGEM-T-MtBL indeed showed 100% stability, pMAV was progressively lost by the recombinant cells, so that at the end of the experiment (50 generations) less than half the population was still recombinant (Fig. 4b).

### Comparison on the four Par systems and proteins

Our results show that *P. haloplanktis* TAC125 possesses a multipartite genome, as it contains two chromosomes and two plasmids. Accurate and coordinated segregation of these genetic elements during cell cycle is due to the presence of a partitioning system in each of them. Partitioning systems are typically found in bacterial chromosomes and in plasmids with a low copy number, and they ensure the distribution of chromosomes and plasmids among daughter cells during replication^44,45^. They are generally composed of three elements: a centromere-like sequence, parS; a centromere-binding protein, ParB; and an NTPase providing energy for the segregation, ParA^46^. *par*S sequence and *par*A and *par*B genes are often organized in a single self-regulated operon. Looking at its main features (summarized in Supplementary Table S7), the partitioning system of pMtBL can be classified as Type Ib, as the NTPase ParA does not contain a helix-turn-helix domain (it is unable to negatively regulate the *parAB* operon transcription). Further evidences support this classification. First of all, orf2 and orf3 are likely to be co-transcribed considering that the predicted SD and start codon of orf3 are superimposed with the end of orf2. Moreover, the lengths of the predicted encoded proteins are compatible with typical type Ib segregation proteins, putative ParA being 213 aa long and ParB 80 aa long^47^. Lastly, the scarcity of close homologues itself is a typical trait of type Ib ParB proteins^47^. The ParAB operons from chromosome I, chromosome II and pMEGA belong to Type Ia system, the most frequent partitioning system in bacterial chromosomes and plasmids^47^. Type Ia system is characterized by the NTPase ParA, which contains a DNA binding domain and is the main transcriptional regulator of the *par* operon, and a well conserved ParB that typically acts as a homodimer and recognizes *parS* sequences generally placed downstream the *par* operon^47^.

Protein similarity searches against the NCBI non-redundant protein database showed that while the most similar sequences to chromosomal ParA and ParB copies are found inside the Pseudoalteromonas genus, the most similar sequences to pMEGA copies are in Vibrio genus. These results suggest that ParA and ParB copies from plasmid and chromosome have a different evolutionary origin.

## Discussion

In this study we have used a combination of NGS and third generation sequencing technologies to resequence the genome of the cold-adapted bacterium *P. haloplanktis* TAC125. NGS sequencing confirmed the already publicly available pMtBL sequence^5^ and all three sequencing technologies revealed the presence of a new plasmid, pMEGA, of more than 60’000 bp. Our resequencing efforts also identified a wrongly assembled tandem repeat in the reference chromosome NC_007481.1. Aside from updating the genome of *P. haloplanktis* TAC125, we performed a comparison of the different sequencing technologies. This comparison allow us to draw some conclusions that might be useful for researchers planning to use third generation sequencing technologies for *de novo* assembly projects.

Third generation sequencing technologies, which produce much longer reads than NGS, are invaluable for disentangle repetitive regions and producing highly continuous and complete genome drafts. Both ONT and PacBio data achieved chromosome level assemblies for *P. haloplanktis* TAC125, including the two reference chromosomes and the novel pMEGA, while Illumina data yielded a much more fragmented genome draft. Although with Illumina data pMEGA was also assembled close to full length, a region of 1.2 kb was assembled highly fragmented into five short contigs due to shared sequence similarity to chromosome NC_007482.1.

Despite the fact that both ONT and PacBio genome drafts were highly continuous and complete, the ONT contigs were less accurate than the PacBio contigs. With 50X PacBio read coverage, PacBio contigs could already achieve consensus accuracy over 99.9%. With increasing PacBio read coverage, the consensus accuracy increased steadily, and already reached 99.97% when the coverage was 100X. In such cases, polishing with Illumina reads was not mandatory and when applied, the process was mainly able to remove the remaining InDels. For the ONT dataset, the sequencing depth had less effect on the final consensus accuracy, which top at 99.18% when the coverage reached 100X and above. Polishing using Illumina data is mandatory for ONT genome drafts when the accepted consensus accuracy is 99.9% or higher. Filtering out low quality ONT reads improved assembly continuity at low coverage and removed assembly artefacts, but the effect on consensus accuracy was marginal and polishing using Illumina data was still needed to reach high accuracy, which was consistent with a previous observation where pre-processing of ONT read based on quality could not remove all errors from the assembly^48^.

The advantage of ONT sequencing was that size selection during library preparation was not mandatory thus it could produce reads much longer than PacBio sequencing, which was essential for producing a highly continuous genome draft at low sequencing depth, as observed in our study. For projects where a highly continuous genome draft is needed but high coverage long read sequencing is not possible, low coverage ONT sequencing (25X) plus polishing with Illumina reads can be a cost-effective solution. Although polishing using Illumina reads could remove many remaining SNPs and InDels, in such genome drafts there could be residual errors that can only be corrected using long reads with sufficient coverage. For projects where both genome continuity and consensus accuracy matters, high coverage long reads (50X PacBio and/or ONT per haploid genome) are needed. When sequencing using PacBio with coverage higher than 100X, Illumina data correction may not be needed if the downstream analysis is not expected to be affected by the residual InDels. When sequencing using ONT, Illumina data correction is always needed to reach the consensus accuracy of 99.9%.

The resequencing of *P. haloplanktis* TAC125 genome using newer sequencing technologies uncovered the presence of a new plasmid, pMEGA. The analysis of *P. haloplanktis* TAC125 plasmids, pMEGA and pMtBL, revealed that they have features similar to other plasmids found in cold-adapted bacteria^37^. Like pMtBL, almost half of the described cold-adapted plasmids are cryptic and small (less than 10 kb)^37^. pMtBL has 4,086 bp and only codes for three ORFs, a hypothetical protein and a putative type Ib partitioning system composed of ParA and ParB proteins, which we demonstrated to ensure pMtBL maintenance after bacterial division. pMEGA codes for 52 ORFs and it might be a mobilizable plasmid, as it contains a putative relaxase. However, we could not identify an *oriT* and further experiments are required to confirm that pMEGA is a mobilizable plasmid. pMEGA, like almost half of the cold-adapted plasmids, carries a RepB replication protein and most of its proteins are involved in plasmid replication and maintenance and amino acid metabolism^37^. pMEGA has two sets of toxin/antitoxin systems and a type Ia partition system suggesting that it is stably maintained after cell division despite its low copy number. Unlike other cold-adapted described plasmids^37^, pMEGA does not contain any resistance to antibiotics or heavy metals, which makes it difficult performing further experiments that require its selection. Among 66 plasmids described in cold-adapted bacteria, only 6 had restriction-modification systems, and all of them belong to type II^37^. However, pMEGA hosts two different restriction-modifications systems, one belonging to type I and the other one to type IV. Interestingly, these two systems are shared with the two most similar plasmids to pMEGA, the unnamed plasmids from *P. nigrifaciens* and *P. arctica*, isolated both from cold marine environments. Other regions of similarity include RepB, ParA and ParB proteins. Despite these similarities, only 35% of pMEGA’s sequence shows homology to *P. nigrifaciens* and *P. arctica* plasmids.

One of the most interesting features of pMEGA is that it contains a DNA PolV, which is an error-prone polymerase that facilitates replication despite DNA damage^40^. We identified a LexA binding site upstream PolV encoding gene, suggesting that its expression is regulated chromosomally by the LexA repressor and that therefore it is induced by the SOS response. Interestingly, *P. haloplanktis* TAC125 contains two DNA PolV operons, one in chromosome II and one in pMEGA, but they do not share a common origin (protein identity of 68%). While pMEGA’s DNA PolV shows high similarity to the DNA PolV found in *P. nigrifaciens* KMM 661 plasmid and in *P. translucida* KK 520 chromosome I, the DNA PolV harboured in chromosome II shares high similarity with *P. nigrifaciens* KMM 661 and *P. translucida* KK520 chromosomal II copies. Closest homologs to *P. haloplanktis* TAC125 DNA PolV chromosomal and plasmid copies are found in Pseudoalteromonas genus, but more distant homologs are also found in Colwellia genus (protein similarity around 67%) and Vibrio (protein similarity around 50%). This observation suggests that DNA PolV is found in other marine bacteria, and in some instances, like in *P. haloplanktis* TAC125, bacteria harbour a copy in a chromosome and a copy in a plasmid. Crucially, it has been suggested that DNA PolV might provide protection against DNA damage caused by the increased UV radiation found in polar regions^37^. Aside from providing protection against DNA damage, the SOS response is a mechanism that elevates the mutation rate, which can increase genetic diversity and facilitate bacterial adaptation to changing environments^49,50^. For example, it has been shown that the mutagenesis induced by an error prone DNA polymerase has been key to facilitate the evolution of legume endosymbionts^51^ or antibiotic resistance^49,52^. *P. haloplanktis* TAC125 genome carries two DNA PolV copies and it is tempting to speculate that this might further enhance its ability to adapt to environmental challenges.

Plasmids frequently carry genes that facilitate the survival of bacteria in challenging conditions^53^, and pMEGA is not an exception. pMEGA encodes several proteins (i.e. DNA PolV, restriction enzymes, proteins involved in metabolism) that might play a crucial role for *P. haloplanktis* TAC125 survival in cold-adapted environments and for its adaptation to environmental changes. pMEGA might contain other interesting proteins, but unfortunately 40% of its ORFs encode unknown proteins, suggesting that further research is needed to fully understand the role of plasmids in adaptation to cold environments.

## Methods

### Library preparation and sequencing

#### Illumina

Genomic DNA from *P. haloplanktis* TAC125 was extracted from 3 ml overnight cultures (2 × 10^9^ cells) grown on minimal marine sea water media supplemented with 0.1% D-Gluconic acid using the DNeasy Blood & Tissue kit (QIAGEN) with some modifications. Briefly, after addition of Proteinase K lysates were incubated at 56 °C for 1 hour. Then, buffer AL was added to the lysate and samples were kept at 70 °C for 10 minutes before adding ethanol. DNA was eluted in 100 μl of EB buffer (QIAGEN). The amount and quality of the genomic DNA (gDNA) extraction was assessed with Qubit Fluorometer dsDNA Broad Range assay and Nanodrop, and integrity of DNA was checked on 0.7% agarose gels. Library preparation and sequencing (HiSeq. 4000, 150 bp paired end reads) was conducted at the Oxford Genomics Centre, Wellcome Centre for Human Genetics.

#### Third generation sequencing

High-molecular-weight (HMW) gDNA from *P. haloplanktis* was isolated as described before^54^ with slight modifications. The same extracted HMW gDNA was used either for ONT GridION X5 and PacBio RSII sequencing. The input gDNA concentration was measured using a Qubit Fluorometer dsDNA Broad Range assay (Life Technologies p/n 32850). A Femto Pulse gDNA analysis assay (AATI p/n FP-1002-0275) was used to assess the DNA integrity and size distribution.

#### ONT GridION X5

The ONT library was constructed following the 1D ligation sequencing kit protocol (Oxford Nanopore p/n SQK-LSK108), without optional shearing. Firstly, 3 μg of gDNA were DNA damage repaired and end repaired using a NEBNext FFPE Repair Mix kit (NEB p/n (M6630) and a NEBNext End Repair/dA Tailing Module kit (NEB p/n E7546), respectively. ONT sequencing adapters were added by ligation, using a Blunt/TA Ligation Master Mix (NEB p/a MO367). The ONT library was then loaded onto a 106 flow cell (Oxford Nanopore p/n R9.4.1), following the manufacturer’s instructions, and sequenced using a GridION X5 machine (Oxford Nanopore). The runtime was 24 hours.

#### PacBio RSII

A SMRT bell library was produced using the SMRTbell Express Template Prep Kit (Pacific Biosciences 101-357-000). 10 μg of gDNA were mechanically sheared to an average size distribution of 15-20 kb, using a Covaris gTube (Covaris p/n 520079). 3 μg of sheared gDNA was DNA damage repaired and end-repaired using polishing enzymes. A ligation reaction was performed to create the SMRT bell template, according to the manufacturer’s instructions. A Blue Pippin device (Sage Science) was used to size select the SMRT bell template and enrich the big fragments beyond 10 kb. The sized selected library was quality inspected and quantified using a Femto Pulse (Agilent) gDNA analysis assay and on a Qubit Fluorometer respectively. A ready to sequence SMRT bell-Polymerase Complex was created using the P6 DNA/Polymerase binding kit 2.0 (Pacific Biosciences p/n 100-236-500) according to the manufacturer instructions. The Pacific Biosciences RSII instrument was programmed to load and sequence the sample on 1 SMRT cell v3.0 (Pacific Biosciences p/n100-171-800), taking 1 movie of 360 minutes. A MagBead loading (PacBio p/n 100-133-600) method was chosen in order to improve the enrichment of the longer fragments.

### *De novo* assembly

Illumina data were assembled using SPAdes (version 3.11.1)^55^.

Due to the high error rate of the ONT and PacBio data, long reads were assembled following the Hierarchical Genome Assembly Process (HGAP)^56^, which relies on a succession of pre-assembly, assembly and consensus polishing steps to generate a genome draft. At the pre-assembly step, error-prone long reads were aligned against each other. Consensus sequences were taken from the alignments to form long and highly accurate reads, which were then assembled during the assembly step. The consensus polishing step is to further reduce the remaining InDel and base substitution errors in the genome assembly. At this step the original set of long reads were aligned back to the assembled contigs. Signal level information per base were taken into account while making the final call of the consensus base. Due to the utilization of signal level information, this step is often sequencer-specific. Nanopolish was developed to polish ONT genome drafts using ONT reads^57^, while PacBio GenomicConsensus tools can polish PacBio genome drafts using PacBio RSII (Quiver, Arrow) and Sequel data (Arrow) (https://github.com/PacificBiosciences/GenomicConsensus). Following this convention, our PacBio data were assembled using HGAP3 in SMRT Analysis 2.3.0 (https://www.pacb.com/documentation/smrt-analysis-software-installation-v2-3-0/), where Quiver was used for the consensus polishing. ONT data were first assembled using Canu (version 1.5)^58^. Afterwards ONT reads were aligned back to the Canu genome draft using bwa mem (version 0.7.15)^59^ for consensus polishing with nanopolish (version 0.10.1)^57^. Polished HGAP3 and Canu assemblies were circularized and trimmed using amos (version 3.1.0)^60^. Circularized and trimmed HGAP3 genome draft was further polished with PacBio reads using the resequencing pipeline (blasr + quiver) within SMRT Analysis 2.3.0. The circularized and trimmed Canu genome draft was again polished with ONT reads using nanopolish, as described above.

To produce the final genome drafts assembled from long read data, the circularized and trimmed contigs were then polished using Pilon (version 1.22)^61^. In detail, Illumina reads were quality controlled (trimmomatic-0.33, adaptor trimming, average quality 20, minimum length 36 nt)^62^, and aligned back to the ONT and PacBio genome drafts using bwa.

For the simulation study, seqtk (https://github.com/lh3/seqtk) was used to sub-sample ONT and PacBio reads to the targeted sequencing depth. Sub-sampled ONT and PacBio reads were assembled and analysed, as described above. NanoFilt^63^ was used to filter out ONT reads with mean quality scores lower than 7. Filtered reads were subsampled and included in the simulation.

### Comparative genomics

For read alignment against the reference genome, bwa mem^59^ was used for Illumina reads, minimap2 for ONT and PacBio reads. Qualimap (2.1.2)^64^ was used to collect alignment based error profiles. Assembled genome drafts were compared to the reference genome (NC_007481.1 and NC_007482.1) using MUMmer (version 3.23)^65^.

BlastN^66^ similarity searches against the NCBI nr/nt nucleotide database were used to identify regions of similarity between pMEGA and other prokaryotic genomes. BlastP^66^ similarity searches against the *P. haloplanktis* TAC125 proteome were used to identify pMEGA proteins homologous to *P. haloplanktis*.

### Plasmid annotation

Plasmids were annotated using Prokka-1.12^67^, RASTtk^68^ and DFAST^69^ annotation pipelines. The final annotation contains genes supported by at least two annotation pipelines. BlastP^66^ similarity searches against the nr protein database and posterior manual curation were used to further annotate the predicted proteins. Searches against the Pfam database were used to functionally annotate the predicted proteins^70^.

CollecTF database^71^ was used to obtain a collection of experimentally validated bacterial LexA binding sites. xFITOM software^72^ together with the collection of bacterial LexA binding sites were used to scan *P. haloplanktis* TAC125 genome (chromosome I, II and pMEGA plasmid) to identify LexA binding sites.

BRIG program was used to visualize pMEGA plasmid, including its comparison to other prokaryotic genomes^73^.

### Bacterial strains, growth media and shuttle plasmids

*E. coli* DH5α was used for cloning and amplification purposes. *E. coli* S17-1(λpir) was used in bacterial conjugations as a donor strain for *P. haloplanktis* TAC125 and Krpl transformations^5^. The psychrophilic cured strain Krpl was used for segregational stability assays, while *P. haloplanktis* TAC125 wt for plasmid copy number (PCN) evaluation. *E. coli* was routinely cultured in LB broth at 37 °C. *P. haloplanktis* TAC125 wt and Krpl were grown in TYP (Bacto Tryptone, 16 g/L; yeast extract, 16 g/L; NaCl, 10 g/L) during interspecific conjugations and preinocula. The plasmid stability and PCN assays were carried out at 15 °C in GG whose composition is reported in Sannino *et al*., 2017. The recombinant strains were cultured in the presence of 100 µg/mL ampicillin.

The recombinant plasmids used in this work are pMAV, CloneQ-*P7-lacZ* and pGEM-T-MtBL. The construction of the first is reported in Sannino *et al*., 2017. CloneQ-*P7-lacZ* was designed during the preparation of a genomic library^13^, while pGEM-T-MtBL was designed using pGEM-T as a backbone^5^. In particular, the whole sequence of pMtBL was cloned into the mesophilic plasmid using XbaI digestion.

### Plasmid copy number quantification

Total DNA was extracted from *P. haloplanktis* TAC125 and Krpl strains using the E.Z.N.A. Bacterial DNA kit (Omega Bio-Tek Inc) following the manufacturer’s instructions. Generally, we collected about 5 × 10^9^ genome copies from 1 OD_600_ pellets of nonrecombinant Krpl according to the following equation:

$${\rm{Mass}}\,{\rm{for}}\,{\rm{one}}\,{\rm{copy}}={\rm{Genome}}\,{\rm{or}}\,{\rm{plasmid}}\,{\rm{size}}\,({\rm{bp}})\times 1.096\times {10}^{-21}\,g/\mathrm{bp}$$

Pure plasmids used in the creation of standard curves were obtained from *E. coli* DH5α with the QIAprep Spin Miniprep Kit (Qiagen) following the manufacturer’s instructions.

#### Validation of the DNA extraction method

Before the plasmid copy number (PCN) quantification in real samples, we first assessed the capacity of the DNA extraction method to efficiently isolate both genomic and plasmid DNA without any preference. To do so we mixed 1 OD_600_ of nonrecombinant Krpl cellular suspensions, corresponding to 5 × 10^9^ genome copies (see above), with defined numbers of copies of pure pGEM-T-MtBL plasmid so to formulate 1:10, 1:50 and 1:100 genome to plasmid ratios. After the DNA extraction we proceeded with the absolute quantification of both the genome and the plasmid in each sample by qPCR using the method of Lee *et al*., 2006. Particularly, pMtBL *orf1* was selected as target for the plasmid quantification and *PSHA_RS10135* as target for the genome detection. *PSHA_RS10135* was selected because a part of it was previously cloned in the CloneQ-*P7*-*lacZ* vector^13^. Hence, qPCR reactions performed on 10-fold serial dilutions of both pure pGEM-T-MtBL and CloneQ-P7-lacZ (from 5 × 10^6^ to 5 × 10^3^ copies) were used to construct the standard curves of orf1 and PSHA_RS10135 genes, respectively. A serial dilution of nonrecombinant Krpl genome was also used to develop a standard curve of PSHA_RS10135 in the chromosome to be sure that the efficiency of the reaction was not affected by the type of the template. Then, the absolute quantity of plasmid and genome in each prepared sample was defined by the interpolation of their Ct value against the corresponding standard curve. Finally, the exact PCN was defined by dividing the measured number of copies of the plasmid by the number of the copies of the genome in each sample. In every case the efficiencies (E) of the standard curves were in the 1.98–2.01 range and they showed a sharp linearity over the chosen dilution series (r^2^ ≥ 0.998). Furthermore, the PCN values of the spiked samples had a linear relationship with the theoretical ratios, indicating that our extraction method did not suffer of a biased affinity for either genomic or plasmid DNA (Supplementary Fig. S8). The primers used for each reaction are reported in Supplementary Table S8.

#### Relative PCN quantification in real samples

For the PCN estimation in unknown samples we extracted the total DNA from 1 OD_600_ cell pellets and performed qPCR reactions. This time a relative method was chosen, as indicated by Škulj *et al*., 2008. Particularly, *PSHA_RS10135* gene was always used to detect the chromosome I in the samples, *PSHA_p00043* was the target for pMEGA quantification, while pMtBL *orf1* was chosen to measure pMtBL PCN (Supplementary Table S8). In these analyses the standard curves were developed using two identical 10-fold serial dilutions of a random real sample (from 6 × 10^3^ to 6 pg of total DNA). In each dilution series either the chromosomal gene or the plasmid gene was the target. For the development of the couples of standard curves the thresholds were set to 0.5 ΔRn and the efficiencies were derived. Then, the relative PCN for each unknown sample was calculated with the following equation:

$${\rm{PCN}}={{\rm{E}}}_{{\rm{c}}}^{{\rm{Ctc}}}/{{\rm{E}}}_{{\rm{p}}}^{{\rm{Ctp}}}$$

where E_c_ and E_p_ are the efficiencies obtained from the standard curves of the amplification of the chromosomal and plasmid genes, respectively, and Ct_c_ and Ct_p_ are the threshold cycles for the two amplicons (chromosomal and plasmid genes) in each sample. In every case the efficiencies (E) of the standard curves were in the 1.98–2.02 range and they showed a sharp linearity over the chosen dilution range (r^2^ ≥ 0.997).

#### qPCR set up with SYBR green dye

qPCR reactions were prepared in 10 µL mixtures containing 1X PowerUp SYBR Green Master Mix (Applied Biosystems) with ROX as passive reference dye and Uracil-DNA glycosidase (UDG) to eliminate contaminations, 400 nM of each primer and 1 µL of sample. Each reaction was performed in triplicate and volumes smaller than 3 µL were not pipetted in the preparation of the mixtures to avoid technical errors. The reaction master mixes were aliquoted in three wells of a reaction plate and the qPCRs were run by a Step One system (Applied Biosystem). The thermal cycling protocol was as follows: UDG activation for 2 min at 50 °C; initial denaturation for 10 min at 95 °C; 40 cycles of denaturation for 15 sec at 95 °C alternated with annealing/extension steps for 1 min at 60 °C. At the end of each reaction a melting curve was obtained to certify the specificity of the chosen primers. Each couple of primers was selected using the free Primer 3 web tool and is reported in Supplementary Table S8.

### RNA extraction and RT-PCR

Total RNA was extracted from *P. haloplanktis* TAC125 cultures using the Direct-zol RNA miniprep plus kit (Zimo-Research) following the manufacturer’s instructions. The quantity and quality of the purified RNA were checked both with a UV spectrophotometer and agarose gel electrophoresis. 1/10^th^ of the extracted material was used as template in the reverse transcription reaction catalyzed by the ProtoScript II Reverse Transcriptase (New England Biolabs). The first strand cDNA from pMtBL *orf2* mRNA was synthetized according to the manufacturer’s instructions using pMtBL_*B7*_rv as primer. Then a standard PCR using pMtBL_*A4*_fw and pMtBL_*B7*_rv primers and *Taq* DNA polymerase (New England Biolabs) was performed to amplify an *orf2* specific region. Templates including total gDNA and total RNA were also used as positive and negative controls, respectively. The sequences of the used primers are listed in Supplementary Table S8.

### Segregational stability assay

The segregational stability of the psychrophilic vectors was assayed during bacterial growth in absence of antibiotic selection. A single colony of the chosen strain was taken from TYP agar selective plates and inoculated in TYP with the antibiotic. After a training of 24 h in GG containing the selective agent, the cells were diluted to 0.1 OD_600_ in fresh medium. Everyday the cultures were diluted in antibiotic-free GG to keep them constantly in the exponential phase (0.2–1.5 OD_600_). At precise intervals of time, culture samples were diluted of a 10^4^ factor and spread onto antibiotic free-TYP agar plates. After two days of incubation at 15 °C at least 30 colonies were selected and replicated on both selective and non-selective TYP agar and incubated again at 15 °C for two days. The ratio of recombinant cells was determined by the comparison of the growing colonies in the two conditions.

The reads generated during this study are available in the European Nucleotide Archive database, under the bioproject PRJEB32057. We deposited the sequence of pMEGA in GenBank, accession number MN400773.

## Supplementary information

Supplementary information

Supplementary Table S3
