# Supplementary material for: New insights on Pseudoalteromonas haloplanktis TAC125 genome organization and benchmarks of genome assembly applications using next and third generation sequencing technologies
Source: Sci Rep. 2019 Nov 11;9:16444. doi: 10.1038/s41598-019-52832-z (PMC6848147; doi:10.1038/s41598-019-52832-z)
Supplement: Supplementary file 1 — Supplementary information [file 41598_2019_52832_MOESM1_ESM.pdf]

New insights on *Pseudoalteromonas haloplanktis* TAC125 genome organization and benchmarks of genome assembly applications using next and third generation sequencing technologies

Weihong Qi*[,#,1], Andrea Colarusso[#,2], Miriam Olombrada[3,4], Ermenegilda Parrilli[2], Andrea Patrignani[1], Maria Luisa Tutino*[,2], Macarena Toll-Riera*[,3,4]

[1] Functional Genomics Center Zurich, ETH Zürich / University of Zurich, Winterthurerstrasse 190, 8057 Zurich, Switzerland

[2] Department of Chemical Sciences, Federico II University of Naples, Complesso Universitario Monte Sant'Angelo, via Cintia, I-80125 Naples, Italy

[3] Department of Evolutionary Biology and Environmental Studies, University of Zurich, Winterthurerstrasse 190, 8057 Zurich, Switzerland

[4] Swiss Institute of Bioinformatics, Quartier Sorge-Bâtiment Génopode, Lausanne 1015, Switzerland

[#]These authors contributed equally to this work

*Corresponding authors: weihong.qi@fgcz.ethz.ch, tutino@unina.it, mtollriera@gmail.com

Supplementary Information

Supplementary Text

pMEGA similarity to *P. haloplanktis* TAC125 chromosomes

Nucleotide similarity to *P. haloplanktis* TAC125 chromosomes is scarce (Supplementary Table S5), and most of it falls in intergenic regions with the exception of two regions. The first region is found in chromosome I, PSHA_p00039-PSHA_p00041 genes show 97.6% identity to chromosome I PSHA_RS02020-PSHA_RS02030 genes. This region is similar to IS*679* insertion sequence[1], which belongs to the IS*66* family[2], and contains three ORFs: *tnpA* (PSHA_RS02020), *tnpB* (PSHA_RS02025) and *tnpC* (PSHA_RS02030). *tnpC* gene has 1,563 bp and its predicted product is presumably a transposase, since it includes a DDE motif (the triad of acidic amino acids that defines a classical transposase active site). *tnpA* (300 bp) and *tnpB* (348 bp) genes function is unknown[2]. The disposition of the three reading frames in IS*679* elements suggests translational coupling. Compared to *tnpA*, *tnpB* is typically found in the translational reading frame -1 and its initiation codon overlaps with the termination codon of *tnpA*. *tnpC* initiation codon is located downstream *tnpB*. A similar organization is present also in chromosome I and in pMEGA analogue regions. Usually, IS*679* members include relatively well-conserved imperfect terminal inverted repeats (IR) of about 20 bp, and putative IR sequences were identified also in the analysed DNA sequences (data not shown).

The second region of similarity is found in chromosome II, PSHA_p00006 displays 96.7% identity to chromosome II PSHA_RS16255. This DNA region contains a non-coding RNA named HEARO (HNH Endonuclease-Associated RNA and ORF) RNAs[3] and a gene coding for an HNH endonuclease (PSHA_RS16255). HNH endonucleases are a family of homing endonucleases, which are frequently embedded within group I and group II introns and are responsible for the transfer of these elements[4]. These enzymes are commonly involved in the transposition of a variety of mobile genetic elements[5]. HEARO representatives are found in species from ten different bacterial phyla, predominantly Firmicutes, Proteobacteria, and Cyanobacteria[3]. This pattern of distribution is a strongly indicative of its function as a selfish genetic element. Thus, HEARO RNA together with its associated HNH endonuclease gene probably form a mobile genetic element. HEARO typically integrates upstream a RUGA motif (ATGA or GTGA)[3]. The comparison of the genomic sequence flanking the HEARO present in pMEGA and in chromosome II with sequences of a corresponding location in different organisms allowed the identification of the conserved RUGA motif at a possible integration site (ATGA) (Supplementary Fig. S9).

Protein similarity searches revealed that pMEGA shows homology to some chromosomal proteins (Supplementary Table S5) aside from the ones mentioned above. Chromosome I hosts a type II toxin-antitoxin system HipA family toxin (homologous to PSHA_p00008), an integrase (homologous to PSHA_p00026), a serine protease (homologous to PSHA_p00029), an endonuclease (homologous to PSHA_p00033) and type I restriction-modification system subunits R, S and M (homologous to PSHA_p00046, PSHA_p00048, PSHA_p00049). However,

homology is low, with a percentage of identity of 37% at maximum. Chromosome II harbours five proteins with homology to pMEGA proteins: chromosome partitioning protein ParA (homologous to PSHA_p00001), a Ton-B receptor (homologous to PSHA_p00010), DNA replication terminus site-binding protein (homologous to PSHA_p00014) and DNA PolV subunit UmuC and UmuD (homologous to PSHA_p00030 and PSHA_p00031), being the maximum percentage of identity 68%. pMEGA and chromosome II share a similar genetic organization (partitioning protein ParA and replication initiator protein), which further supports the unidirectional mechanisms of chromosome II replication due to the clear plasmidic origin of the abovementioned protein functions[6].

pMEGA similarity to other bacteria

Shared regions of similarity between pMEGA, *P. arctica* and *P. nigrifaciens* contain the ParA (PSHA_p00001) and ParB (PSHA_p00002) proteins, DNA replication terminus site-binding protein (PSHA_p00014), an hypothetical protein (PSHA_p00025), an integrase (PSHA_p00026), Type I restriction-modification system (PSHA_p00045-PSHA_p00049), the restriction endonuclease Mrr (PSHA_p00050), an hypothetical protein (PSHA_p00051) and the RepB family plasmid replication initiatior protein (PSHA_p00052). Despite these regions are found both in P*. arctica* and *P. nigrifaciens*, *P. nigrifaciens* shows a higher percentage of identity with pMEGA, suggesting that *P. nigrifaciens* plasmid is the closest related sequence to pMEGA. Additionally, *P. nigrifaciens* also shows homology to the RepB family plasmid replication initiator protein (PSHA_p00027) and to the DNA PolV operon (PSHA_p00030-PSHA_p00033) and *P. arctica* to NYN domain-containing protein (PSHA_p00004) and Type II toxin-antitoxin HipBA system (PSHA_p00008-PSHA_p00009).

Taking advantage from the high level of nucleotide sequence conservation amongst the pMEGA plasmid and *P. arctica* and *P. nigrifaciens* plasmids, the multiple alignment of the nucleotide sequence encompassing the functions involved in replication initiation (*repB*) and plasmid partitioning (*parAB*), allowed us to make some hypothesis concerning regulation of these functions in the psychrophilic plasmids. *repB* and *parAB* operon are transcribed by two divergent promoters (likely overlapping) located in the 279 bp long region (this distance is 270 bp and 269 bp in *P. nigrifaciens* and *P. arctica* plasmids, respectively) which separates RepB and ParA translational start sites. This organization suggests a common (negative) regulation of both promoters by the binding of ParA when its concentration rises, due to a higher plasmid copy number[7]. pMEGA RepB is a Rolling Circle Replication (RCR) initiator protein, belonging to the Rep_3 superfamily (PF01051). Its capacity to bind specific DNA sequences (the bind site) and to exert topoisomerase-like function allows the enzyme to cleave a specific DNA sequence (the nick site) and to release a 3'-OH free end while it remains bound to the 5'-P end by a phosphotyrosine link[8]. A careful inspection of the sequence downstream the *repB* gene highlights the presence of two direct repeats, located 45 base pairs from a potential hairpin forming sequence, which may represent the nick site[8].

References

1. Gourbeyre, E., Siguier, P. & Chandler, M. Route 66: investigations into the organisation and distribution of the IS66 family of prokaryotic insertion sequences. *Res. Microbiol.* **161**, 136–43 (2010).
2. Han, C. G., Shiga, Y., Tobe, T., Sasakawa, C. & Ohtsubo, E. Structural and functional characterization of IS679 and IS66-family elements. *J. Bacteriol.* **183**, 4296–304 (2001).
3. Weinberg, Z., Perreault, J., Meyer, M. M. & Breaker, R. R. Exceptional structured noncoding RNAs revealed by bacterial metagenome analysis. *Nature* **462**, 656–9 (2009).
4. Stoddard, B. L. Homing endonuclease structure and function. *Q. Rev. Biophys.* **38**, 49 (2006).
5. Burt, A. & Koufopanou, V. Homing endonuclease genes: the rise and fall and rise again of a selfish element. *Curr. Opin. Genet. Dev.* **14**, 609–15 (2004).
6. Médigue, C. *et al.* Coping with cold: the genome of the versatile marine Antarctica bacterium Pseudoalteromonas haloplanktis TAC125. *Genome Res.* **15**, 1325–35 (2005).
7. Ebersbach, G. & Gerdes, K. Plasmid Segregation Mechanisms. *Annu. Rev. Genet.* **39**, 453–479 (2005).
8. Ruiz-Masó, J. A. *et al.* Plasmid Rolling-Circle Replication. *Microbiol. Spectr.* **3**, (2015).
9. De Coster, W., D'Hert, S., Schultz, D. T., Cruts, M. & Van Broeckhoven, C. NanoPack: visualizing and processing long-read sequencing data. *Bioinformatics* **34**, 2666–2669 (2018).

Supplementary Figures

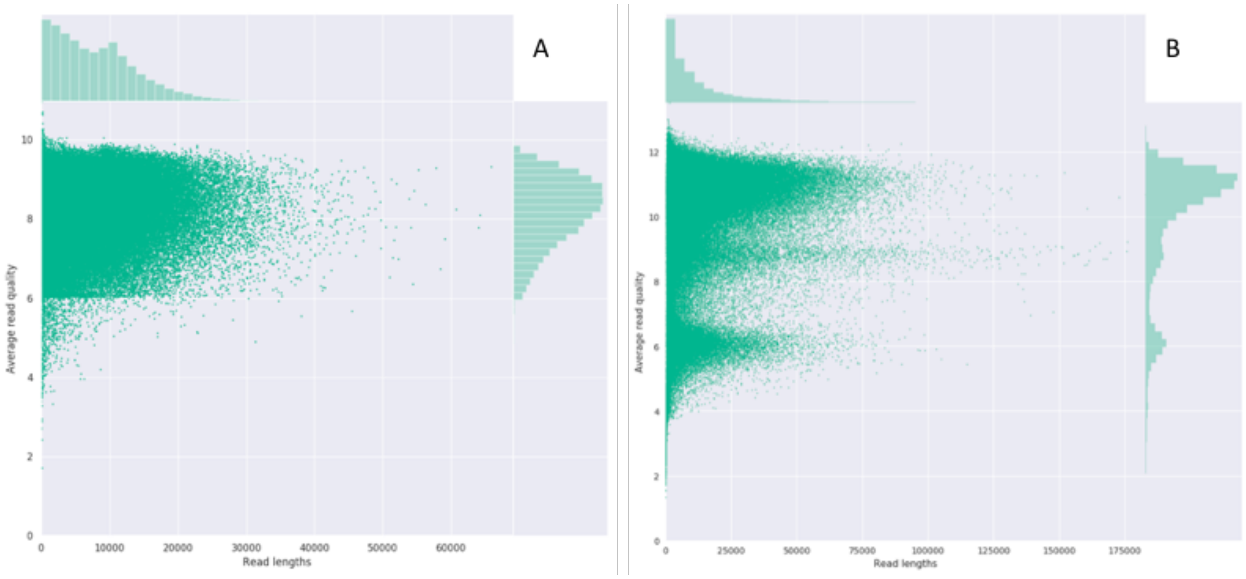

Supplementary Figure S1. Multi-panel plots of average base quality per read vs. read length. (A) PacBio raw reads. (B) ONT raw reads. Within each plot, the read length histogram is shown on the top panel, the histogram of average base quality per read on the side panel. Plots were generated using NanoPlot[9]. PacBio read quality distribution had one peak centred around the average quality score, while ONT read quality distribution had multiple peaks and higher variability.

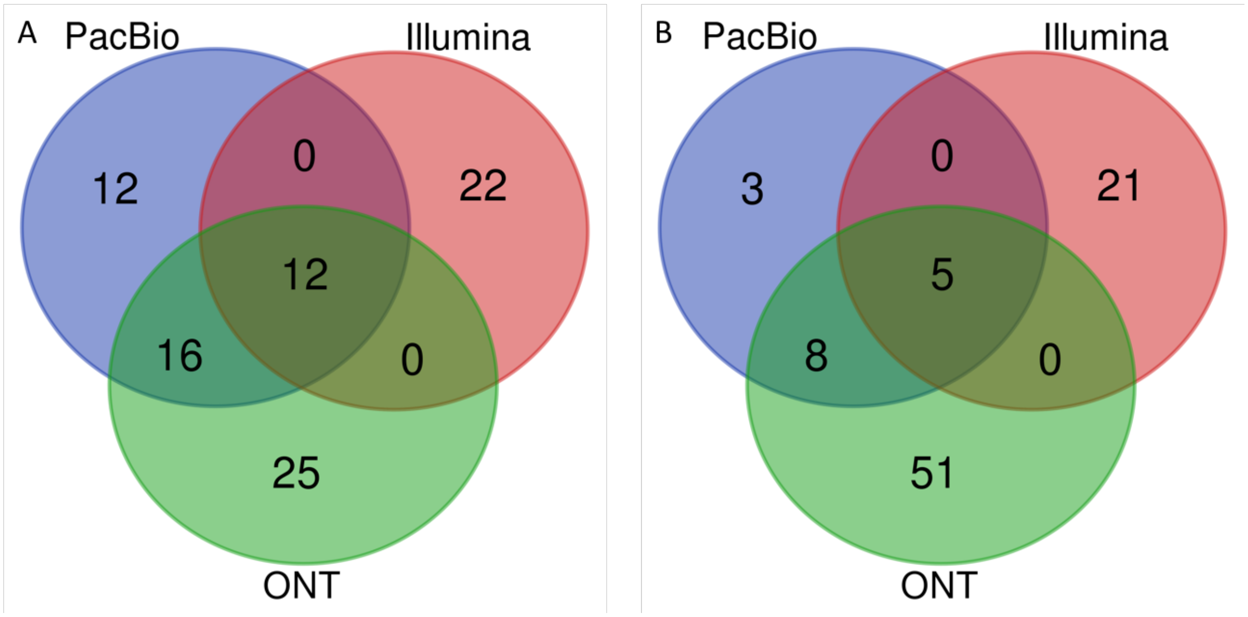

Supplementary Figure S2. Venn diagram showing the amount of residual SNPs (A) and InDels (B) that overlapped between the drafts assembled from the three technologies. The total number of residual InDels per draft is lower than that listed in Table 2 because homopolymer insertions were collapsed in drawing the Venn diagram, but counted as multiple insertions.

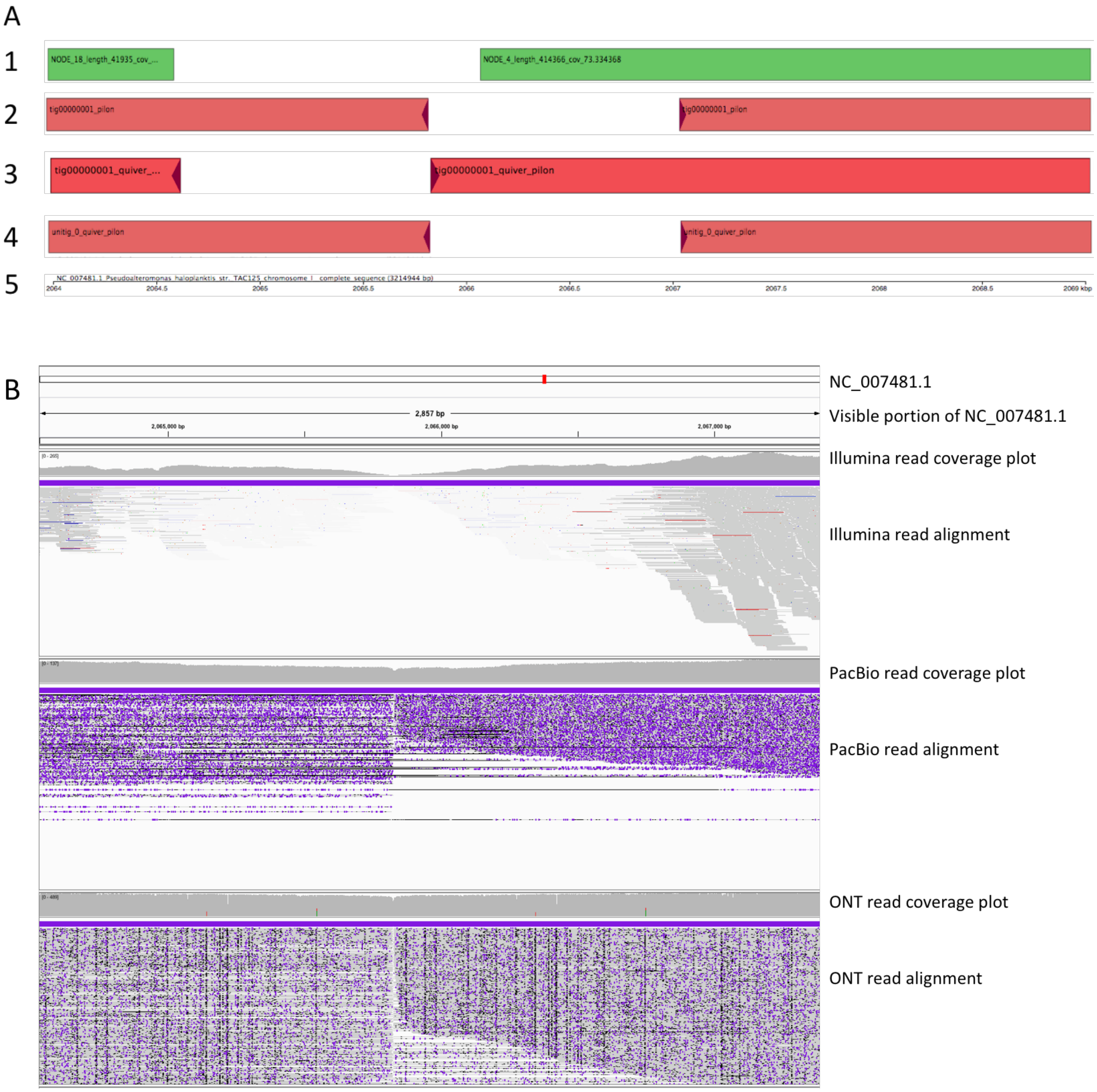
A
1
NODE_18_length_41935_cov_...
NODE_4_length_414366_cov_73.334368
2
tig00000001_pilon
tig00000001_pilon
3
tig00000001_quiver_...
tig00000001_quiver_pilon
4
unitig_0_quiver_pilon
unitig_0_quiver_pilon
5
NC_007481.1 Pseudoalteromonas haloplanktis str. TAC125 chromosome I, complete sequence (3214944 bp)
2064
2064.5
2065
2065.5
2066
2066.5
2067
2067.5
2068
2068.5
2069 kbp
B
2,857 bp
2,065,000 bp
2,066,000 bp
2,067,000 bp
NC_007481.1
Visible portion of NC_007481.1
Illumina read coverage plot
Illumina read alignment
PacBio read coverage plot
PacBio read alignment
ONT read coverage plot
ONT read alignment

C

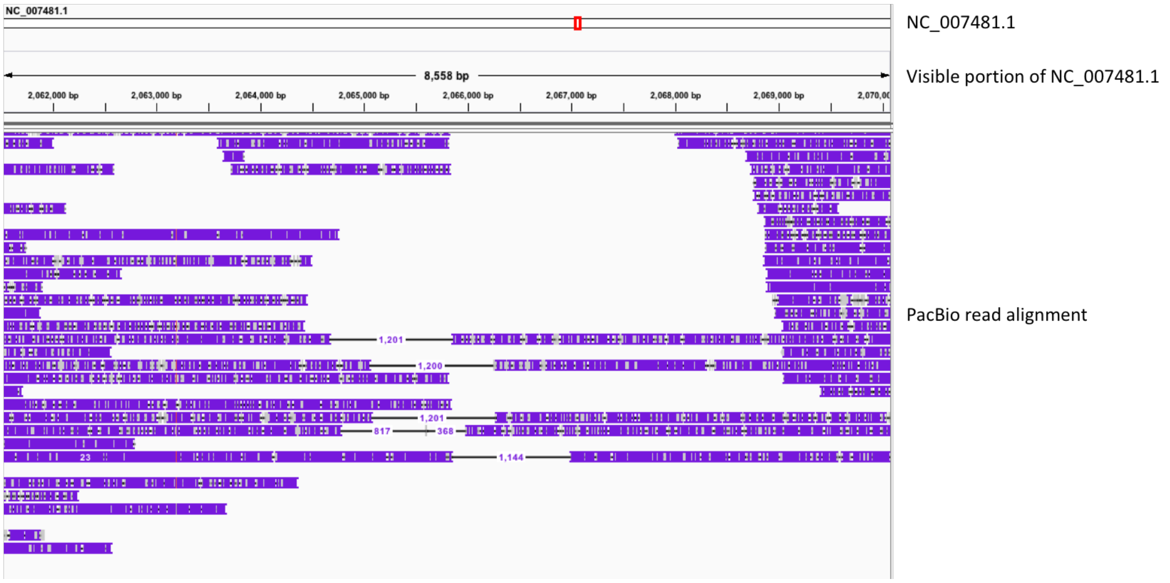

Supplementary Figure S3. Re-sequencing of *P. haloplanktis* TAC125 genome identified one assembly error in the reference chromosome NC_007481.1, where the sequence between 2,064,625 and 2,065,827 was wrongly assembled twice to form a tandem repeat. (A) Alignment of assembled contigs across the miss assembled region (NC_007481.1: 2,064,625-2,065,828-2,067,030). Illumina (track 1), ONT (track 2) and PacBio contigs (track 3 by Canu, track 4 by HGAP3) all contained only one copy of the sequence. ONT and PacBio contigs aligned splitted across this region (track 5), which were covered by two Illumina contigs. (B) Alignment of Illumina paired-end reads, PacBio reads and ONT reads to the reference chromosome NC_007481.1, around the tandem repeat region. With all three sequencing technologies, a coverage drop within this region was observed, suggesting a false assembly event. The middle of this region seemed fragile and created hard breaks during sequencing. Most PacBio and ONT reads ended or started around there. But a small amount of PacBio long reads did sequence through this region and the split alignment across the tandem repeat (C) further suggested there should be only one copy of the sequence, not two.

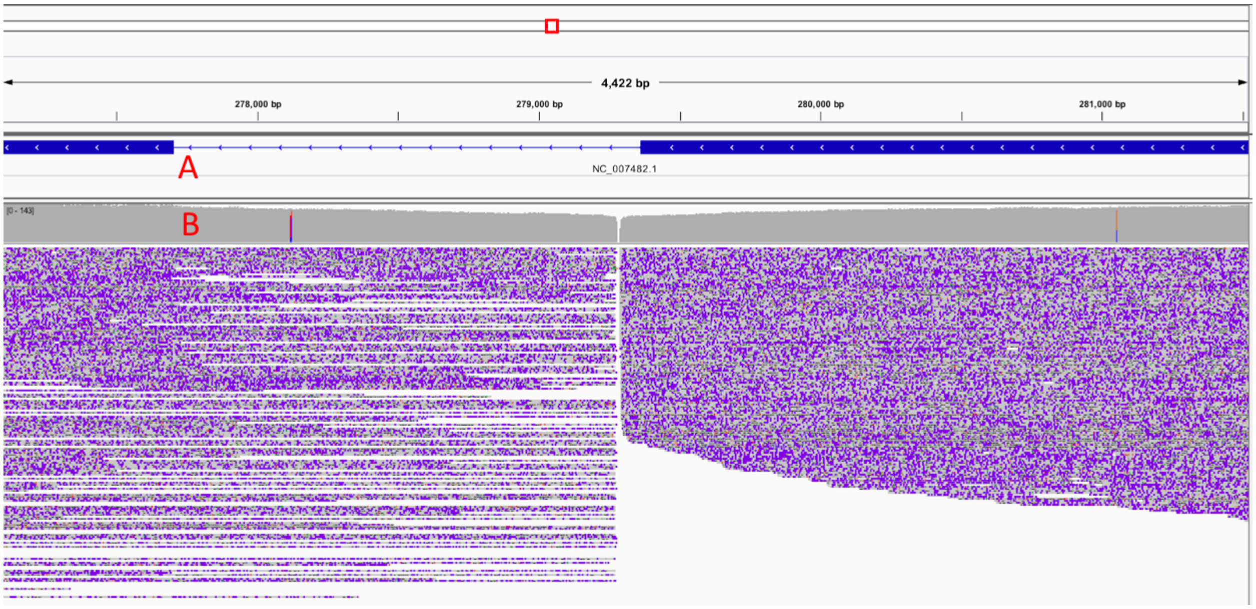

Supplementary Figure S4. The error in the final ONT draft, where the 1644 bp sequence between 560,857-562,502 on chromosome NC_007482.1 was assembled tandemly in the ONT contig, with a 10 bp novel sequence fragment inserted in between. (A) The reference chromosome NC_007482.1 was aligned split across the miss assembled region. (B) Alignment of PacBio reads across this region showed also a coverage drop, suggesting a false assembly event. No PacBio reads aligned through the 10 bp novel sequence region, further suggested the tandem repeat was an error.

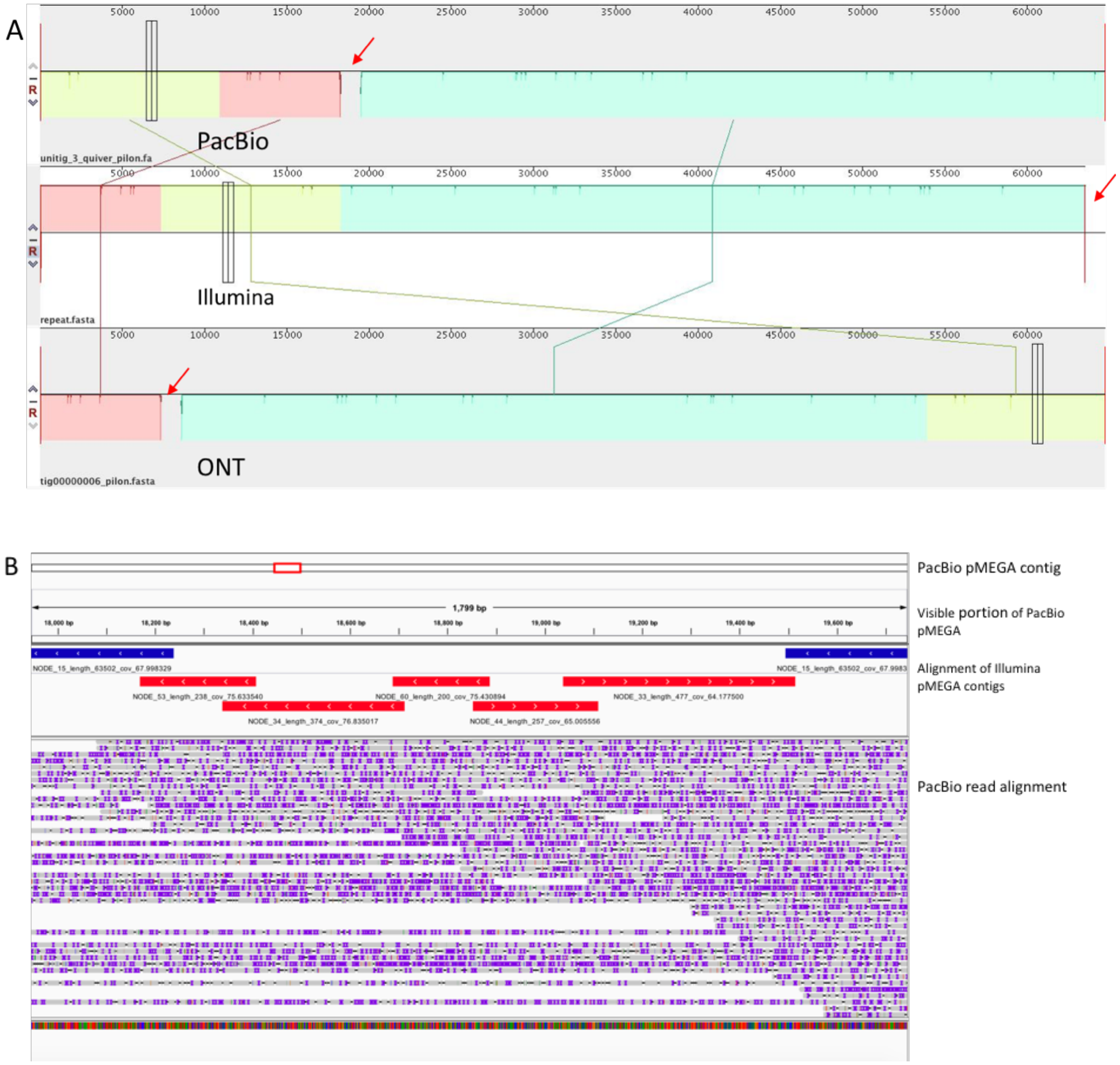

Supplementary Figure S5. Both PacBio and ONT data assembled pMEGA into one circularized contig, while the Illumina pMEGA consisted of six contigs and was not circularized. (A) Multiple alignment of pMEGA contigs assembled using three different sequencing technologies. Both PacBio and ONT contigs harboured a 1.2 kb region, as highlighted by the red arrows, which was missing in the longest Illumina contig, but covered by five short contigs, as shown in (B). In this figure, the longest Illumina pMEGA contig aligned splitted against the PacBio pMEGA contig (dark blue lines). The junction of the split alignment was tiled by other five short Illumina contigs (red lines). PacBio read alignment along the PacBio pMEGA contig revealed the presence of long reads spanning the 1.2 kb region, which helped to resolve the repetitiveness and yielded chromosome level assembly.

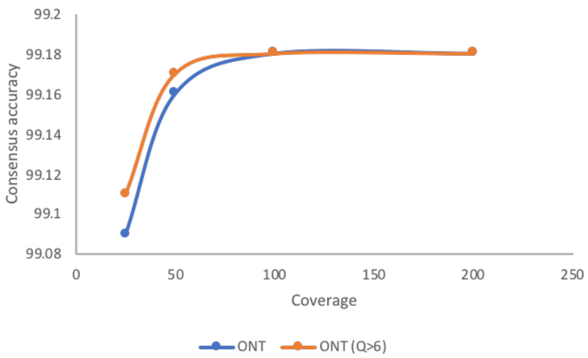

Supplementary Figure S6. Effect of ONT read quality filtering on the consensus accuracy of Canu assemblies of ONT reads.

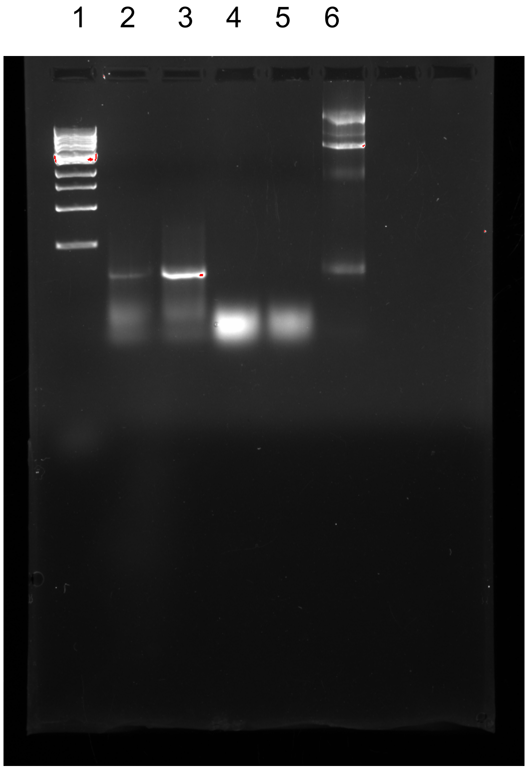

Supplementary Figure S7. Full-length gel corresponding to the gel displayed in Figure 3b.

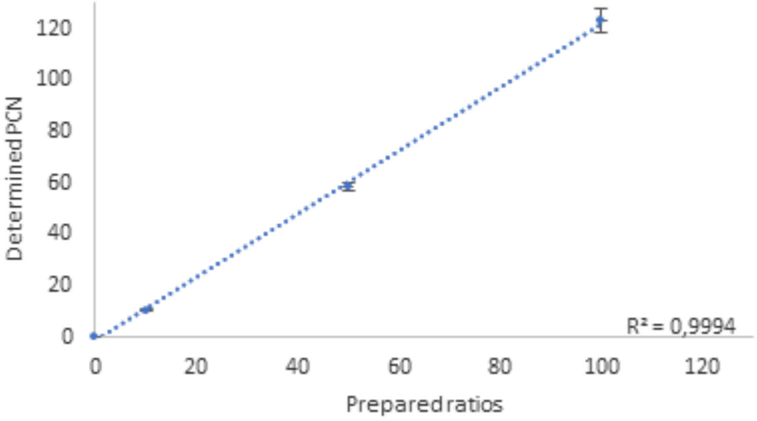

Supplementary Figure S8. Assessment of the total DNA extraction kit. Mixtures of non-transformed cells and purified plasmid pGEM-T-MtBL were prepared in precise ratios. After total DNA recovery, the PCN was defined via qPCR with the absolute method.

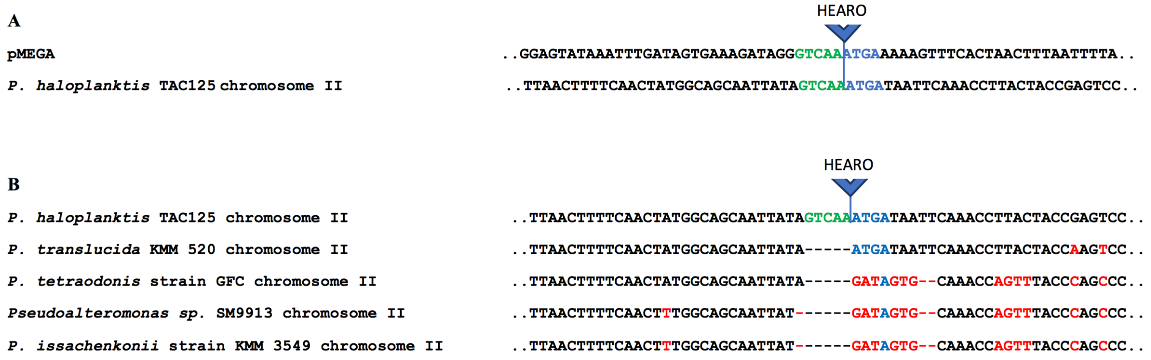

Supplementary Figure S9. Comparisons of the genomic sequence flanking the HEARO location in pMEGA and *P. haloplanktis* TAC125 chromosome II (A) and the genomic sequences of chromosome II of four different *Pseudoalteromonas* strains homologous to *P. haloplanktis* TAC125 chromosome II (B). In green the first five nucleotides of conserved HEARO RNA are highlighted. Blue letters designate the conserved RUGA motif at a possible integration site. Red letters/dashes highlight the differences in the alignment with respect to *P. haloplanktis* TAC125 chromosome II.

Supplementary Tables

Supplementary Table S1. Residual SNPs and InDels that were supported by all three technologies

Chromosome	SNPs/InDels
NC_007481.1	228517GA 1579547GT 228493GC 2063196CT 1675783GT 1674145GA 343585GT 228434AG<br>1168599G. 159039.T 536067A. 1303681G.
NC_007482.1	326161CA 343586GA 343587GT 343588TC<br>87873A.

Supplementary Table S2. Assembly outcomes of the pMEGA by all technologies

	ONT [1]	PacBio [1]	Illumina [2]
Num. contigs	1	1	6
Total length (bp)	64,757	64,758	65,048
N50 (bp)	64,757	64,758	63,502
GC%	38.60	38.61	38.47
Num. N's	24	0	0
Num. N's per 100 kb	37.06	0	0

[1] Circularized, trimmed and polished assemblies
[2] Raw assembly

Supplementary Table S3. Influence of sequencing coverage and ONT read quality on Canu assembly* of ONT and PacBio reads.

	ONT			ONT (Mean Quality > 6)			PacBio		
	Num. contigs	N50 (Mb)	Total. Size (Mb)	Num. contigs	N50 (Mb)	Total. Size (Mb)	Num. contigs	N50 (Mb)	Total. Size (Mb)
25X	4	3.240	4.103	3	3.171	3.923	6	1.220	3.881
50X	3	3.243	3.980	3	3.237	4.018	3	3.205	3.949
100X	5	3.238	4.150	3	3.253	4.078	3	3.226	3.965

200X	6	3.243	4.178	3	3.256	4.081	3	3.228	3.973
500X	3	3.271	4.110	3	3.271	4.110	NA	NA	NA

* Raw assembly stats, without circularization and trimming

Supplementary Table S4. Effects of polishing strategies on the final consensus accuracy of long read assemblies.

	ONT		PacBio	
Polishing datasets (tools, and coverage)	Illumina reads (Pilon, 259X)	Illumina reads (Pilon, 259X) and ONT reads (Nanopolish, 573X)	Illumina reads (Pilon, 259X)	Illumina reads (Pilon, 259X) and PacBio reads (Quiver, 195X)
Residual SNPs	277	53	40	40
Residual InDels	237	87	29	24

Supplementary Table S5. pMEGA nucleotide and protein similarity searches against *P. haloplanktis* TAC125 (NC_007481.1, NC_007482.1), *P. nigrifaciens* strain KMM 661 plasmid (CP0110381) and *P. arctica* A 37-1-2 plasmid (CP011027.1) (excel file).

Supplementary Table S6. pMEGA nucleotide similarity searches against the NCBI nucleotide collection (nr/nt)

Description	Query cover	E-value	Identity	Accession
Pseudoalteromonas arctica A 37-1-2 plasmid unnamed, complete sequence	36%	0.0	97%	CP011027.1
Pseudoalteromonas nigrifaciens strain KMM 661 plasmid, complete sequence	34%	0.0	98%	CP011038.1
Pseudoalteromonas translucida KMM 520 chromosome I, complete sequence	13%	0.0	93%	CP011034.1
Pseudoalteromonas arctica A 37-1-2 chromosome I, complete sequence	12%	0.0	91%	CP011025.1

Supplementary Table S7. Main features of the four partitioning systems responsible for *P. haloplanktis* TAC125 chromosomes I and II, pMEGA and pMtBL plasmids maintenance.

	Chromosome I	Chromosome II	pMEGA	pMtBL
ParA	261 aa	412 aa	401 aa	213 aa
ParB	308 aa<br>Spo0J family	320 aa<br>Spo0J family	360 aa<br>SopB family	80 aa<br>Ribbon H-H
Classification	Type Ia	Type Ia	Type Ia	Type Ib

Supplementary Table S8. Primers used in this work.

Primer name	Sequence (5' – 3')	Purpose, position
pMtBL_*A4*_fw<br>pMtBL_*B7*_rv	ATGAGCTGGGCTATATGC<br>AACCTCCTGATACAAATC	RT-PCR of pMtBL *orf2* mRNA,<br>1398 – 1564 [a]
*Prom7*_fw<br>*Prom7*_rv	CCTTTATTCAGCGTGTTGGCGAGC<br>GTTATCAGGGTCGGGCGTATCGG	qPCR of *PSHA_RS10135*,<br>2168812 – 2168846 [b]
pMEGA_*CDS40*_fw<br>pMEGA_*CDS40*_rv	AACTGACTGTGGTGCTCTTC<br>ACTGGTCCCTATTTGTTTATGCT	qPCR of pMEGA PSHA_p00043,<br>47314 – 47392 [c]
pMtBL_*orf1*_fw<br>pMtBL_*orf1*_rv	AATGACGCTGGACTGAGAA<br>CCTGGCGAACTCCTGAAA	qPCR of pMtBL *orf1*,<br>527 – 596 [a]

fw: forward. rv: reverse.
[a], the coordinates are referred to pMtBL sequence (AJ224742, NCBI)
[b], the coordinates are referred to TAC125 chromosome I sequence (NC_007481.1, NCBI).
[c], the coordinates are referred to pMEGA sequence.
